# Corneal complication after femtosecond laser-assisted cataract surgery: A case report

**DOI:** 10.1097/MD.0000000000024013

**Published:** 2021-01-15

**Authors:** Edyta Chlasta-Twardzik, Anna Nowińska, Edward Wylęgała

**Affiliations:** Chair and Clinical Department of Ophthalmology, Faculty of Medical Sciences in Zabrze, Medical University of Silesia in Katowice; Ophthalmology Department, Railway Hospital, Katowice, Poland.

**Keywords:** cataract surgery, corneal complication, corneal confocal microscopy, femtosecond laser, femtosecond laser-assisted cataract surgery

## Abstract

**Rationale:**

The femtosecond laser LDV Z 8 is unique, and the only femtosecond laser used in ophthalmic microsurgery, which is characterized by low-energy near-infrared (1030 nm) femtosecond single pulses in the nano-Joule range and a very high repetition rate in the MHz range. To the best of our knowledge, this is the first description of unintentional partial corneal incision in the anterior part of a stroma by the femtosecond laser.

**Patient concerns:**

A 79-year-old patient was referred to our clinic for cataract surgery. On admission, we diagnosed mature cataract of the left eye and pseudophakic of the right eye. The patient was qualified for the Femtosecond Laser-Assisted Cataract Surgery (FLACS). Unexpectedly during FLACS procedure after lens fragmentation the surgeon observed unexpected paracentral incision in the cornea.

**Diagnosis:**

The corneal incision line between 4 to 8 o’clock was observed. In vivo confocal microscopy was performed to assess the morphology and depth of the corneal wound. The corneal incision covered the epithelium, Bowman's membrane and stroma of the cornea reached a depth of 336 um.

**Interventions:**

The patient was under increased ophthalmologic controls; follow up with typical ophthalmic examinations and confocal microscopy was performed.

**Outcomes:**

In 2-year follow-up period, this complication had no effect on postoperative visual function, the patient had no visual problems and obtained the final BCVA 5/5. There is no dislocation of the lens in 2 years follow-up.

**Lessons:**

Low pulse energy and high pulse frequency in the LDV Z8 causes a low traumatization of tissues. In a 2-year follow-up, the corneal incision line could be observed on the slit lamp examination without long-term visual consequences of this complication. In our opinion, the most likely cause of this complication was human error and lack of communication between medical personnel. The operation team should be alert and great caution must be exercised during the procedure and check the laser settings parameters carefully each time.

## 1 Introduction

Cataract is a common eye disease and in addition to uncorrected refractive errors remains the leading cause of reversible blindness worldwide.^[1,2]^ Cataract surgery is considered to be one of the most effective and the safest medical procedures used in the world, which has been confirmed in clinical studies. Cataract surgery is the most commonly performed surgery in the world. One of the cataract surgery techniques that Charles Kelman developed in the 1970s, phacoemulsification, has become nowadays the standard surgical technique used in the developed world.^[3]^ The femtosecond laser (FL) is one of the newest generation of lasers used in ophthalmology. Femtosecond laser technology was first introduced in corneal refractive surgery for performing LASIK flaps. The introduction of the FL into cataract surgery was in 2009 year by Zoltan Nagy.^[4]^ Recently, dynamic development and improvement of the cataract surgery technique has been observed. In the cataract surgery, FL systems are used for performing anterior capsulotomy, lens fragmentation using different patterns, clear corneal incisions, and arcuate incisions. The Femto LDV Z8 (Ziemer Ophthalmic Systems AG, Port, Switzerland) is a high frequency FL system for corneal surgery, corneal-refractive surgery as well as cataract surgery. The Femto LDV Z 8 is unique among femtosecond cataract laser systems. It is the only FL used in ophthalmic microsurgery, which is characterized by low-energy near-infarted (1030 nm) femtosecond single pulses in the nano-Joule range and an extremely high repetition rate in the MHz range. This laser enables an even more precise and accurate cataract removal procedure with 10 times less energy consumption. This concept was originally developed by Ziemer Ophthalmic Systems firstly for corneal surgery and later adapted for cataract surgery applications. LDV Z 8 has a new liquid–patient interface thanks to that a good transmission of the laser beam is provided. The precise location of the ocular surfaces during surgery is possible thanks to integrated high-definition ocular coherence tomography. Additional advantage of the FL is a minimal intraocular pressure increase during procedure.^[5,6]^

## 2 Case report

A 79-year-old woman was admitted for cataract surgery. The patient reported a progressive visual acuity deterioration in the left eye lasting for several months. Examination on admission revealed, pseudophakic of the right eye and mature cataract of the left eye. Best corrected visual acuity (BCVA): right eye 5/10 (cc +1.0Dsph); left eye 2/25. Intraocular pressure (IOP): right eye 19 mm Hg; left eye −19 mm Hg. Autorefractometry: right eye +0.25Dsph −0.25Dcyl ax. 5∗; left eye −3.5Dsph −0.75D cyl ax. 87∗. Keratometry (Anterior Segment Optical Coherence Tomography; Tomey CASIA 2) right eye: 45.61/47.01D; left eye: 46.5/47.0D. Axial length (IOL Master Carl Zeiss Meditec, Inc, Dublin, CA): right eye: 22.31 mm; left eye −22.49 mm. Pachymetry (Anterior Segment Optical Coherence Tomography; Tomey CASIA 2) right eye: 556 um; left eye: 559 um. Corneal endothelial cell density (Topcon SP-3000P) right eye: 2288/mm^2^; left eye 2750/mm^2^. Fundus examination of the right and left eye was irrelevant. Based on medical history and the clinical picture, pseudophackic of the right eye and mature cataract of the left eye were diagnosed. After a qualifying examination and consultation with an anesthetist, the patient was qualified for a surgery under local anesthesia the following day. The patient was qualified for the Femtosecond Laser Assisted Cataract Surgery (FLACS). The FLACS procedure was performed using the Femto LDV Z8 (Ziemer Ophthalmic Systems AG, Port, Switzerland). The procedure was performed under topical anesthesia. Initially, the suction ring of a disposable liquid–patient interface was precisely docked onto the patient's eye and centered over the limbus. After the suction ring was filled with a balanced salt solution (BSS), the handpiece, which is attached to an articulating arm of the laser system, was docked over the corneal apex. The ocular structures were imaged by the integrated optical coherence tomography (OCT) system. Treatment parameters were customized individually to the patient by using the laser platform settings wizard. Femtosecond laser-assisted cataract surgery has been started. Unexpectedly, to the surgeon's surprise after the lens fragmentation instead of capsulotomy the laser made a paracentral incision in the corneal stroma between 4 and 8 o’clock. The experienced surgeon noticed the incision in the corneal stroma immediately and stopped the treatment. After this complication, the surgeon decided to stop use the FL for cataract surgery and perform the further steps of the procedure by traditional manual phacoemulsification technique. Typical small incision phacoemulsification procedure with implantation of the intraocular lens (IOL) into the lens capsule was performed. The next steps of manual phacoemulsification were uncomplicated. Postoperative therapy consisted of a topical steroid (0.1% dexamethasone), antibiotic (levofloxacin) and a nonsteroidal anti-inflammatory drug-NSAID (0.1% nevanac) were prescribed for the patient. Levofloxacin eye drops five times a day for 7 days; dexamethasone eye drops five times a day for 7 days, followed four times a day for 7 days, followed three times a day for 7 days; nepafenac 0.1% (Nevanac 1 mg/mL) eye drops three times a day for 3 weeks.

**Figure 1:**
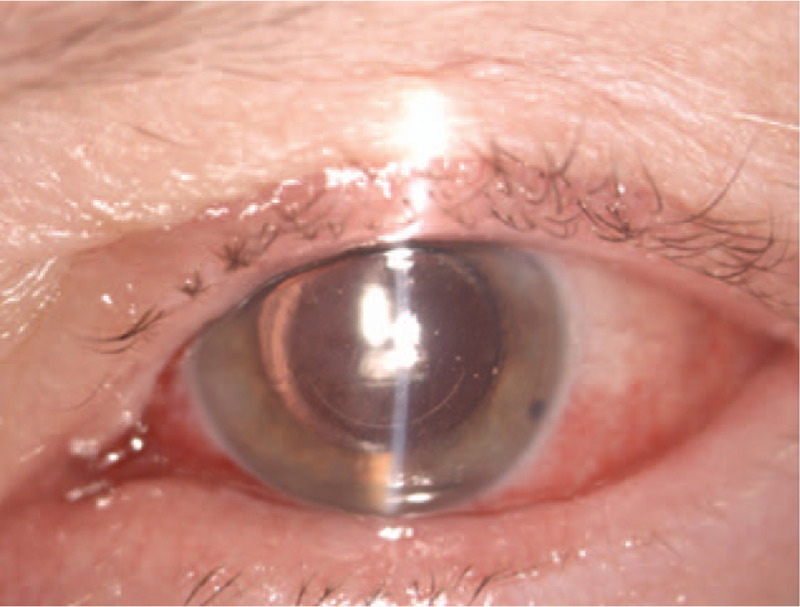
Color photography of the left eye. A first postoperative day. Slit lamp image showing the corneal incision line between 4 and 8 o’clock, a slight subconjunctival effusion, a transparent cornea with a slight corneal edema.

**Figure 2:**
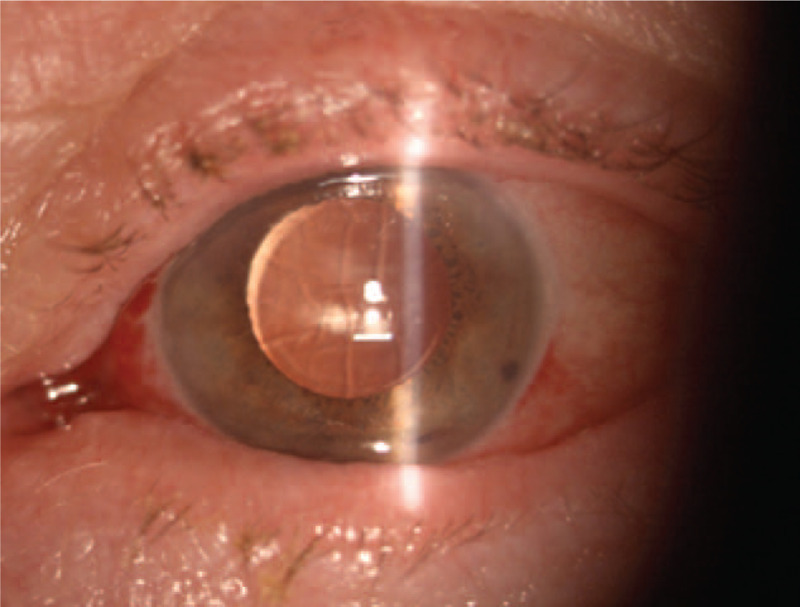
Color photography of the left eye. A first postoperative day. Slit lamp image showing a slight subconjunctival effusion, slight corneal edema with Descemet's folds and a well-centered IOL. The dotted line shows the range of corneal incision line.

On the first postoperative day, the parameters of the left eye were as follow autorefractometry: +0.75D sph −2.75Dcyl axis 72∗; BCVA: 5/12; IOP: 14 mm Hg; keratometry: 46.1/46.8D; central corneal thickness 668 um. On the slit lamp examination, a slight subconjunctival effusion in the left eye was observed resulting from the vacuum applied during the procedure (Fig. 1). A transparent cornea with slight edema and Descemet's folds were seen. Additionally, the corneal incision line between 4 and 8 o’clock was observed (Fig. 2). No signs of inflammation within the left eye were perceived. The patient was discharged home on the first day after surgery in good overall and local condition and with appropriate instructions. She reported for a follow-up 7 days after hospital discharge.

Postoperative follow-up 7 days after surgery

- autorefractometry of the right and the left eye: +0.25Dsph −0.25Dcyl 5∗; +0.5Dsph −2.0Dcyl 84∗, respectively.
- the best corrected visual acuity: 5/10 (cc +1.0Dsph) in the right eye; 5/8 cc +0.25Dsph −1.75Dycl axis 95∗ in the left eye.
- IOP of the right and the left eye: 15 mm Hg; 14 mm Hg
- keratometry of the right and the left eye: 45.6/47.0; 45.7/46.9D
- central corneal thickness of the right and left eye: 556 um; 593 um

**Figure 3:**
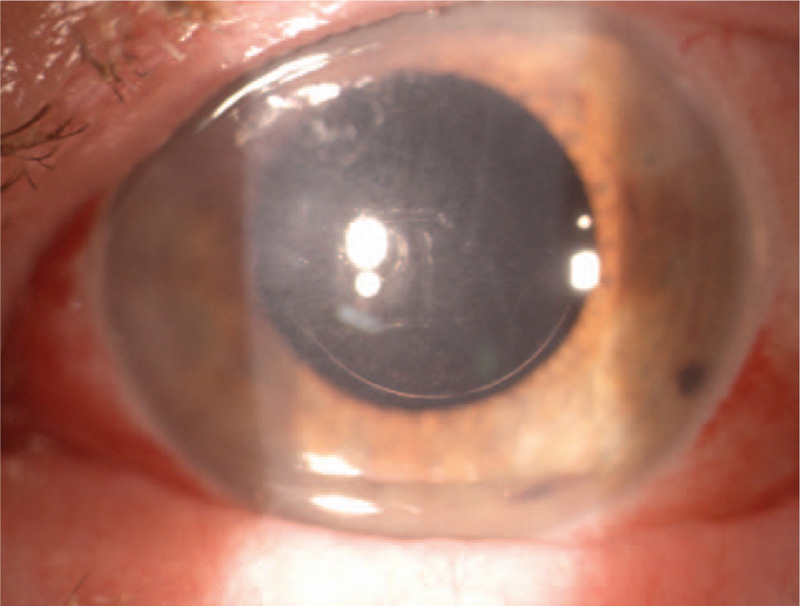
Color photography of the left eye. Seven days after surgery. Slit lamp image showing the corneal incision line between 4 and 8 o’clock, a whole transparent cornea, a lasting slight subconjunctival effusion, a proper axial position of the IOL.

**Figure 4:**
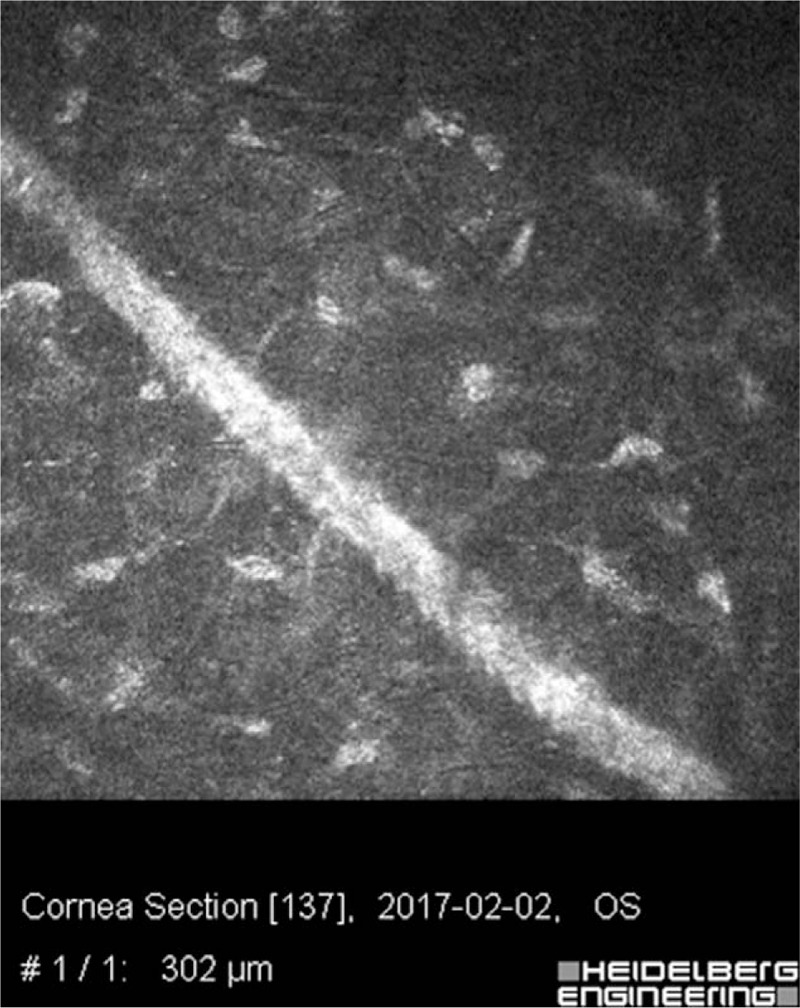
In vivo laser confocal microscopy image of the left eye. High reflectivity of the corneal scar in the stroma. No inflammation cells around wound.

On the slit lamp examination of the left eye the corneal incision line between 4 and 8 o’clock was observed. A whole transparent cornea was perceived. The anterior and posterior segment of the left eye did not revealed signs of inflammation. The intraocular lens was found to remain in an axial position, within the capsular bag (Fig. 3). To assess the morphology of the incision scar and its depth additional imaging of the corneal morphology and integrity in vivo was performed by the used of confocal microscopy. The corneal incision covered the epithelium, Bowman's membrane and stroma of the cornea reached a depth of 336 um (Fig. 4).

In a follow-up examination 1 month after the surgery:

- autorefractometry: +0.25Dsph −0.25Dcyl 5∗; in the right eye; +0.25Dsph −1.75Dcyl axis 90∗, in the left eye.
- the best corrected visual acuity: 5/10Dsph (cc +1.0Dsph) in the right eye; 5/6 cc −1.5Dcyl axis 95∗ in the left eye.
- IOP: right eye 14 mm Hg; left eye 13 mm Hg.
- keratometry of the right and the left eye: 45.4/46.8D; 46.6/47.0D
- central corneal thickness of the right and left eye: 556 um; 563 um

**Figure 5:**
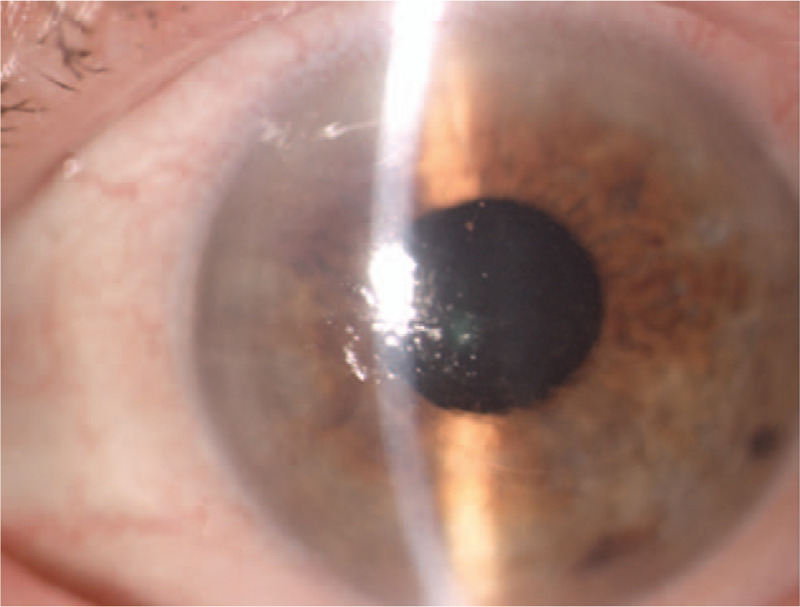
Color photography of the left eye. One month after surgery. Slit lamp image showing the corneal incision line between 4 and 8 o’clock, a stable anterior segment of the eye with a proper axial position of the IOL.

**Figure 6:**
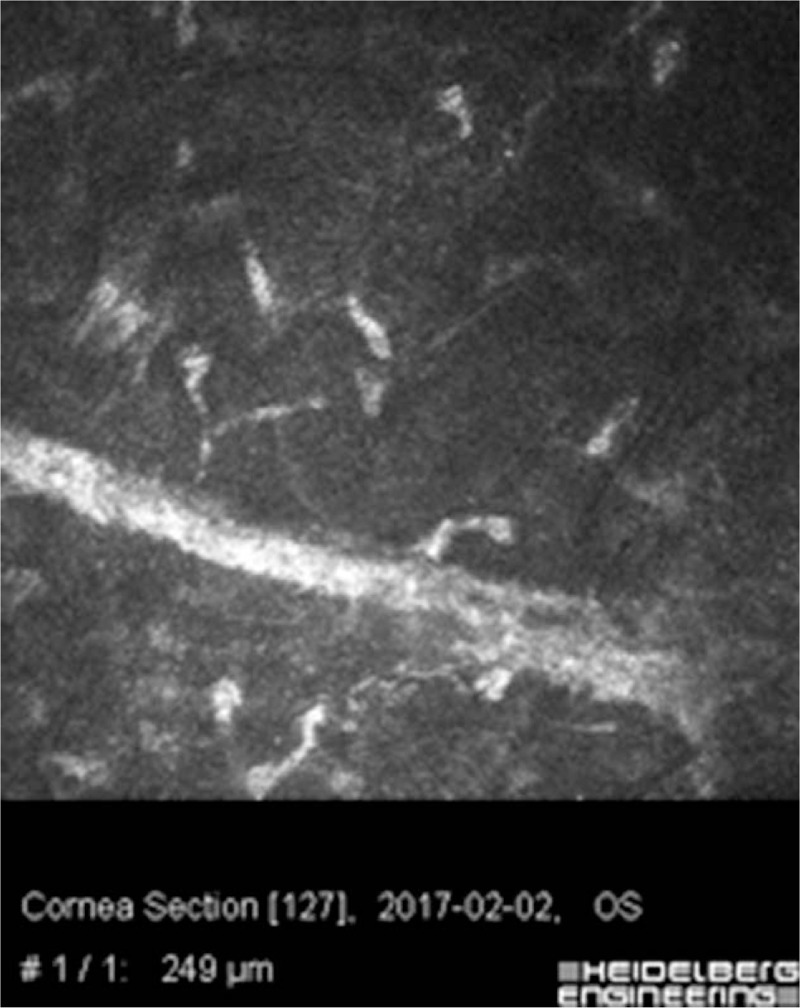
In vivo laser confocal microscopy image of the left eye. High reflectivity of the fully healed corneal scar reaching the depth of 336 um in the corneal stroma. No inflammation cells around wound.

On the slit lamp examination, the corneal incision line between 4 and 8 o’clock was still observed, besides this the anterior segment of the left eye was stable with transparent optical media and proper centration of the intraocular lens (Fig. 5). The fundus image of the left eye was normal. Postoperative refractometry revealed slight postoperative induced astigmatism consequent to the presence of a corneal incision that slightly changed keratometry of the cornea. Confocal microscopy examination in vivo showed a fully healed the incision in the corneal stroma (Fig. 6).

**Figure 7:**
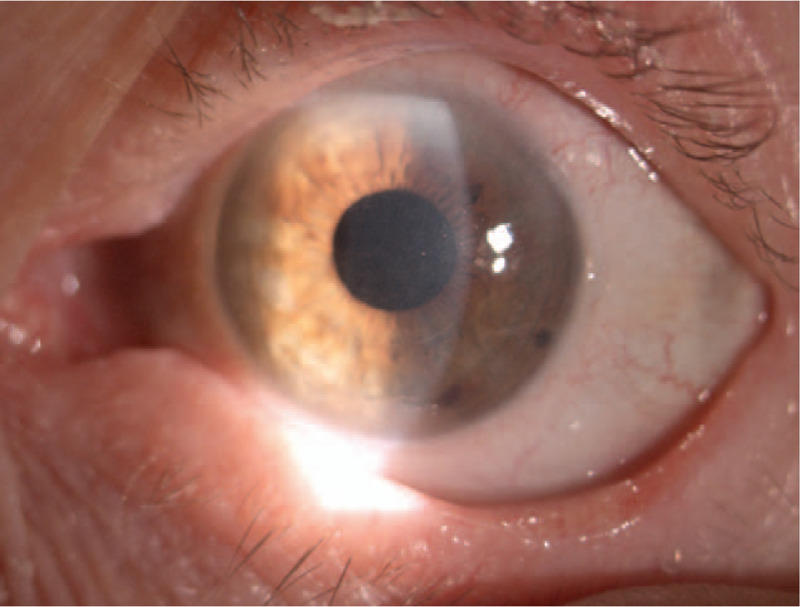
Color photography of the left eye. Two years after surgery. Slit lamp image showing the corneal incision line between 4 and 8 o’clock, a transparent optical media, a proper centration of the intraocular lens.

**Figure 8:**
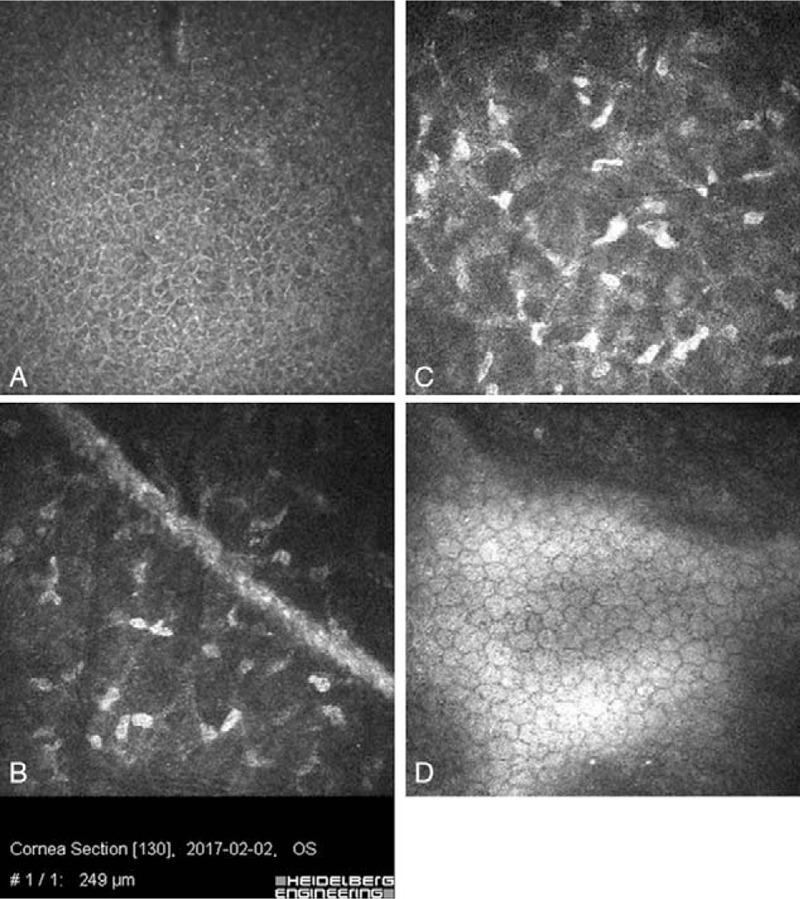
In vivo laser confocal microscopy images. (A) Normal superficial epithelial cells layer with compact organized cells. (B) Clearly visible hyper-reflective scar after corneal incision in the stroma. No inflammation cells around wound. (C) In mid-stroma, normal keratocyte nuclei. (D) Normal appearance of corneal endothelium.

In the 2 years follow-up examination, the patient had no visual problems because of the small partial corneal incision. On the slit lamp examination, the corneal paracentral incision line between 4 and 8 o’clock in the left eye was still observed. The anterior segment of the left eye was stable with transparent optical media and a proper, central position of the IOL in the capsular bag of the left eye was revealed (Fig. 7). The fundus image of the left eye was normal. Confocal microscopy in vivo was performed. The morphological picture remained the same as in the study 1 month after surgery (Fig. 8A–D). Postoperative astigmatism in the left eye was slightly higher after the surgery but it did not affect the patient's subjective feelings. Anterior and posterior corneal curvature was slightly changed in comparing to the parameters before the surgery. The finally BCVA in the left eye was 5/5. The patient did not complain about any of the eye symptoms in 2 years period of observation.

In a follow-up examination 2 years after the surgery:

- autorefractometry: +0.25Dsph −0.25Dcyl 5∗; in the right eye; +0.25Dsph −1.5Dcyl axis 95∗, in the left eye.
- the best corrected visual acuity: 5/10 (cc +1.0Dsph) Dsph in the right eye; 5/5 cc −1.5Dycl axis 95∗ in the left eye.
- IOP: right eye 15 mm Hg; left eye 15 mm Hg.
- keratometry of the right and the left eye: 45.4/46.8D; 47.8/46.1D
- central corneal thickness of the right and left eye: 556 um; 552 um

## 3 Discussion

The FL has been long used in ophthalmic surgery, its use in cataract surgery is relatively new technology and still become more popular.^[7]^ In the literature, there have been many publications about the advantages of using the FL-assisted in cataract surgery compared to the conventional phacoemulsification cataract surgery.^[4,8–11]^ The usage of the FL in cataract surgery is safe, effective, and repeatable, that has been well documented in the literature.^[12,13]^ Despite many advantages, this technique also carries the risk of intraoperative complications characteristic of using a FL in cataract surgery, such as: suction break, capsule tags and bridges, conjunctival redness, or hemorrhage and miosis. The Femto LDV Z8 (Ziemer Ophthalmic Systems AG, Port, Switzerland) emits waves with a length of 1053 nm so that they are not absorbed by transparent optical media. Due to the highly targeted scanning pulse in near infrared, the laser beam can be focused in the area of 3 μm with an accuracy of 1 μm.^[14]^ An additional advantage of LDV Z 8 is integrated optical coherence tomography (OCT) system located in the handpiece therefore, thanks to that we get accurate imaging of the anterior segment structures of the eye.

To the best of our knowledge, this is the first description of unintentional partial corneal incision in the anterior part of a stroma by the FL. The Femto LDV Z8 is used for corneal surgery, corneal-refractive surgery as well as cataract surgery. In our opinion, the most likely cause of this complication that occurred during cataract surgery in presented case report was human error. The range for human error is quite large. A crucial step in FL treatment is a proper docking as well as accurate and proper setting of laser parameters individually for each patient. In our opinion, there may have been no corrected position of the capsulorexis incision on one of the scans when setting the laser parameters directly before the procedure. During the procedure, the laser setting parameters are corrected by the assistants. Lack of communication between medical personnel resulted in this undesirable event. One should be alert and great caution during the procedure and check the laser settings parameters carefully each time. The most common cause of intraoperative complications is human error, an example of which is the presented case report.

Due to the ultra-short duration of a single light pulse of 10^–15^ s, the energy demand for tissue destruction is reduced, which allows an increase in the safety margin and reduction of unintentional destruction of surrounding tissues.^[14,15]^ Shorter pulse duration reduces the energy expenditure needed to achieve a specific goal, which is important in cataract surgery, during which we save the cornea, iris, Zinn's ligaments and lens capsule.^[15]^ In the presented case report, despite the occurrence of an unintended complication which was a laser corneal incision, the LDV Z 8 femto laser turned out to be extremely safe. On slit lamp examination after surgery the corneal incision line could be observed, but the patient did not complain of any other side effects and visual problems in this eye. The eye was healing fast and no further complications occurred. Our case report indicate that the healing time involved in visual acuity is fast in the first few postoperative days and there were no visual consequences of this complication in long-term follow-up period. The finally BCVA in the left eye was 5/5.

Nagy ZZ et al described a similar complication of corneal incision by a laser during FL-assisted cataract surgery. The endothelial cell layer was hit and cut by the laser during capsulotomy creation in 3 highly hyperopic eyes with a shallow anterior chamber. The patient interface flattened the cornea during the FL procedure, the safety distance from the endothelium was insufficient in these eyes with shallow anterior chamber. Probably it was caused by the lack of an integrated OCT system.^[16]^ In our presented case report, the corneal incision by the laser was located in the anterior part of the cornea, included epithelium, Bowman's membrane to the 336 um depth in the stroma. In our case similar to those 3 described by Nagy ZZ et al there were no long-term visual consequences of this complication and all patients reached the best corrected visual acuity 5/5. In a follow-up examination 2 years after surgery the corneal incision line could be observed. In three patients described by Nagy ZZ et al 1 year after surgery the endothelial incision line could be observed.

During the cataract surgery when the corneal cut is too central, it can cause surgically induced astigmatism, when the corneal wound is created manually by the surgeon as well as by the laser. In our case report as a result of the paracentral partial corneal incision, the corneal curvature was slightly changed, and surgical astigmatism was induced. Changes in corneal curvature were quantified using a corneal topographic map with the use of anterior segment optical coherence tomography (CASIA 2, Tomey) before and after cataract surgery. The patient did not experience any other side effects resulting from the paracentral incision in the cornea. The patient did not complain of any other sight problems in this eye and achieved final best corrected visual acuity 5/5.

## 4 Conclusion

To conclude, most complications are predictable and largely preventable. That is why, the surgical increased vigilance is so important and needed during the surgical procedure and especially during the learning curve period. The most common cause of complications that occur in medicine is human error, that is why a communication between medical personnel is so important, but it is often overlooked. It is important to be very careful when programming parameters for surgery in the laser menu and be alert when operating the laser following the stated recommendations, so that intraoperative complications can be avoided. A crucial step in FL treatment is a proper docking. Treatment parameters are customized individually to each patient by using the laser platform settings wizard. Low pulse energy and high pulse frequency in the LDV Z8 causes a low traumatization of tissues. In the presented case report the unintentional laser cut in the corneal stroma in the left eye had no effect on postoperative visual function, the patient had no visual problems and obtained the final BCVA 5/5.

## Author contributions

**Conceptualization:** Edyta Chlasta-Twardzik.

**Data curation:** Edyta Chlata-Twardzik.

**Formal analysis:** Edyta Chlasta-Twardzik, Anna Nowińska.

**Funding acquisition:** Edward Wylęgała.

**Investigation:** Edyta Chlasta-Twardzik.

**Methodology:** Edyta Chlasta-Twardzik, Anna Nowińska.

**Project administration:** Edyta Chlasta-Twardzik.

**Resources:** Edyta Chlasta-Twardzik.

**Software:** Edyta Chlasta-Twardzik.

**Supervision:** Anna Nowińska, Edward Wylęgała.

**Validation:** Edyta Chlasta-Twardzik.

**Visualisation:** Edyta Chlasta-Twardzik.

**Writing – orginal draft:** Edyta Chlasta-Twardzik.

**Writing – review & editing:** Anna Nowńska Edward Wylęgała.

## Abbreviations

Abbreviations: BCVA = best-corrected visual acuity, BSS = balanced salt solution, FL = femtosecond laser, FLACS = femtosecond laser-assisted cataract surgery, IOL = intraocular lens, IOP = intraocular pressure, LASIK = laser in situ keratomileusis, NSAID = nonsteroidal anti-inflammatory drug, OCT = optical coherence tomography.
